# Weakly Supervised Depth Estimation for 3D Imaging with Single Camera Fringe Projection Profilometry

**DOI:** 10.3390/s24051701

**Published:** 2024-03-06

**Authors:** Chunqian Tan, Wanzhong Song

**Affiliations:** College of Computer Science, Sichuan University, Chengdu 610065, China; 2021223040117@stu.scu.edu.cn

**Keywords:** fringe projection profilometry, depth estimation, weakly supervised learning

## Abstract

Fringe projection profilometry (FPP) is widely used for high-accuracy 3D imaging. However, employing multiple sets of fringe patterns ensures 3D reconstruction accuracy while inevitably constraining the measurement speed. Conventional dual-frequency FPP reduces the number of fringe patterns for one reconstruction to six or fewer, but the highest period-number of fringe patterns generally is limited because of phase errors. Deep learning makes depth estimation from fringe images possible. Inspired by unsupervised monocular depth estimation, this paper proposes a novel, weakly supervised method of depth estimation for single-camera FPP. The trained network can estimate the depth from three frames of 64-period fringe images. The proposed method is more efficient in terms of fringe pattern efficiency by at least 50% compared to conventional FPP. The experimental results show that the method achieves competitive accuracy compared to the supervised method and is significantly superior to the conventional dual-frequency methods.

## 1. Introduction

Fringe projection profilometry (FPP) [1] is widely used for 3D imaging because of its high accuracy and speed. FPP usually employs phase-shifting profilometry (PSP) [2,3] or Fourier transform profilometry (FTP) [4] to retrieve the continuous phase and determine the corresponding point-pairs between the camera image and projector pattern. It then uses triangulation to achieve 3D reconstruction.

FTP only needs to take one frame of fringe images to recover the continuous phase. However, with high speed, the continuous phase cannot be extracted correctly with FTP when the object surface changes abruptly or has discontinuous areas. PSP is usually used more widely to ensure the 3D reconstruction accuracy. PSP projects a group of phase-shifting sinusoidal fringe patterns onto the object’s surface, and the camera captures the deformed fringe image. Height information of the object’s surface is naturally encoded into the deformed fringe image. The employment of phase-shifting improves measurement accuracy. However, the use of multiple images also dramatically limits the speed [5]. In addition, the phase-shifting method assumes that the object to be measured remains stationary during each 3D imaging so that motion artifacts will affect the 3D imaging accuracy [6,7].

Balancing 3D imaging speed and accuracy, a common practice is projecting two sets of three-step phase-shifting fringe patterns (referred to as dual-frequency PSP). Thus, one frame of depth maps can be achieved using six frames of fringe images. In some special cases, for instance, the reflectivity of the object surface is uniformly distributed, and the background light outside the object in the scene is fixed. The number of fringe images required for one 3D imaging can be reduced from six to four or five [8]. Without loss of generality, the number of fringe images required for one 3D imaging is usually six with conventional FPP technology. In FPP, the higher the period-number of the high-frequency fringe patterns, the higher the 3D imaging accuracy. When dual-frequency PSP is employed, the highest period-number of fringe patterns is generally fewer than 32 [9].

With the development of artificial intelligence, there has been much work conducted to combine deep learning with FPP in recent years [10,11,12,13,14,15,16]. Van der Jeught and Dirckx [17] proposed a deep-learning-based method for extracting the depth map from a single fringe image. Their experiment was conducted on simulated datasets. Nguyen et al. [18] utilized end-to-end networks to predict depth maps from one frame of fringe images [19]. In their study, three networks, FCN, AEN, and UNet, were compared, and the results showed that UNet performed best. Later, Nguyen et al. [20] investigated the impact of different structured light patterns on the accuracy of monocular depth estimation. The examined input patterns included two high-frequency fringe patterns (vertical and slanted at 45°), two distinct grid patterns with various levels of brightness, a speckle pattern, and a low-frequency fringe pattern. Five coarse-to-fine output depth maps were generated in the decoder stage for supervision. The results demonstrated that the speckle pattern and low-frequency fringe pattern exhibited poorer performance, while there was no significant performance difference among the other patterns. Nguyen et al. [21] employed sinusoidal patterns with period-numbers of 61, 70, and 80 as the RGB channels of a color image, and a network was trained to predict the wrapped phases. Huang et al. [22] obtained the fringe orders using the three-wavelength heterodyne method. They selected a 64-period wrapped phase and two fringe images (period-numbers of 53 and 58) as the input to train a network that could predict the fringe orders corresponding to the wrapped phase. Zheng et al. [23] built one digital twin of a real FPP system using the 3D rendering software Blender. In their study, simulated FPP fringe images were used to train the network for depth estimation from single fringe images. Simulated fringe images are free of motion blur, and many fringe images can be synthesized quickly. Compared with the real FPP system, this method saves many workforce and time costs. However, the model trained on the simulated data has limited generality on real FPP systems. Wang et al. [24] utilized Blender to construct a simulated single-camera FPP system, employing virtual objects from the dataset [25]. Their FPP dataset is collected through the adjustment of various parameters, including the projector’s power, fringe periods, the angle between the camera and projector, and the rotation of fringes, among others. Their study performed experiments on the UNet and pix2pix networks, introducing a novel loss function that combined the structural similarity (SSIM) index and Laplace operator. The outcomes indicated that the UNet network outperformed the others in terms of depth estimation. Wang et al. [26] proposed a depthwise separable Dilation Inceptionv2-UNet to improve the accuracy of 3D measurement from a single-shot fringe pattern. Their experiments were conducted on simulated datasets.

The aforementioned methods of deep-learning-based estimation depth from a single fringe image are desired for single-shot 3D imaging, but they require accurate depth maps as the learning targets. These methods are referred to as fully supervised methods. For fully supervised methods, building a training dataset with ground-truth depth maps is time-consuming and still difficult in many scenarios, such as dynamic scenes. There is an urgent need for unsupervised or weakly supervised methods that do not require ground-truth depth maps as labels.

Fan et al. [27] used unsupervised learning for depth estimation from simulated dual-frequency fringe images. A fringe projection model was established to synthesize new fringe images from the estimated depth and projection pattern. The difference between the synthesized and input fringe images formed the supervision signal to guide the convergence of neural networks. The projection model of [27] was a simplification of real FPP systems. Moreover, the period-numbers of the dual-frequency in their study were 10 and 29, which limited the 3D imaging accuracy.

This study presents a weakly-supervised framework for depth map prediction from fringe images of single-camera FPP. The neural network is trained using the supervisory signals from a one-period phase map and high-frequency fringe images. Depth maps are no longer needed as the labels. After training, the network can predict the depth map from three frames of high-frequency fringe images. In summary, the main contributions of this study are:

(1) A new depth estimation scheme from fringe images is proposed. Compared to the fully supervised method, this scheme no longer requires depth maps as the labels. This change makes this deep-learning-based scheme easier to employ in various FPP application scenarios.
(2) A combination of the self-supervised and weakly-supervised signals is designed to guide the training of the depth estimation network.
(3) Depth maps can be extracted from three frames of 64-period fringe images during inferencing.
(4) Experimental results indicate that the weakly supervised method has competitive depth accuracy compared to the supervised method and is significantly superior to the conventional dual-frequency PSP method, especially in noisy scenes.

Section 2 introduces details of the proposed methodology for weakly supervised depth estimation. The experimental results and discussion are presented in Section 3. Section 4 and Section 5 summarize the conclusions and future work.

## 2. Method

**Figure 1:**
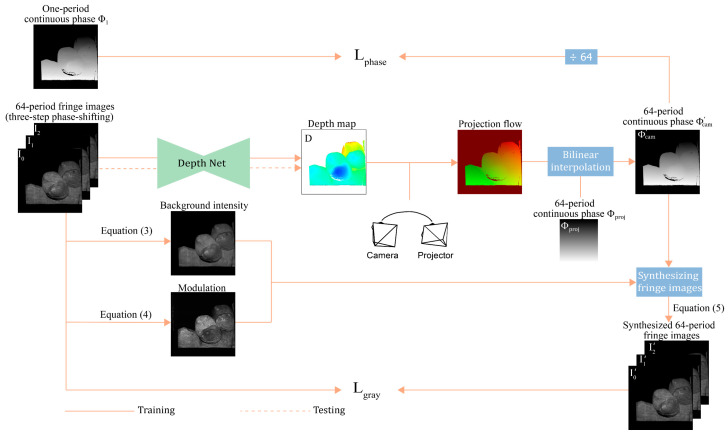
Overview of the proposed weakly-supervised depth estimation framework. A neural network is trained to estimate the depth from three high-frequency fringe images. The self-supervised signal $L_{gray}$ and the weakly-supervised signal $L_{phase}$ replace the labels of depth maps used by existing fully-supervised methods. During testing, the network can recover the depth map from three frames of fringe images.

This study employs weakly supervised deep learning to train a network for predicting the depth map from three frames of fringe images for FPP. The framework of this method is depicted in Figure 1.

First, grayscale consistency constraint on high-frequency fringe images is employed to guide the network’s training. The background intensity A and the modulation B are calculated from three-step phase-shifting high-frequency fringe images ($I_{0}$, $I_{1}$, and $I_{2}$). The three fringe images are fed into a neural network to estimate a depth map D. With the predicted depth D, the relative pose of the camera and projector, and the continuous phase $\Phi_{proj}$ of high-frequency patterns on the projector plane, one continuous phase map $\Phi_{cam}^{\prime }$ of high frequency in the camera view is generated. The continuous phase $\Phi_{cam}^{\prime }$ is modulated into three synthesized fringe images ($I_{0}^{\prime }$, $I_{1}^{\prime }$, and $I_{2}^{\prime }$) by coupling it with background light intensity A and modulation B. The differences between the fringe images ($I_{0}$, $I_{1}$, and $I_{2}$) and the synthesized fringe images ($I_{0}^{\prime }$, $I_{1}^{\prime }$, and $I_{2}^{\prime }$) are used to build the loss function for training the network. This is performed in a self-supervised manner using only high-frequency fringe images.

Depth prediction networks trained with only grayscale consistency error cannot work correctly. Phase consistency is introduced as an additional supervised signal, which measures the error between one-period phase $\Phi_{1}$ and one-period phase $\Phi_{1}^{\prime }$ ($\Phi_{1}^{\prime }=\Phi_{cam}^{\prime }$/64). The total loss function is formulated as follows:

(1) $$Loss=\alpha L_{gray}+\beta L_{phase},$$

where $L_{gray}$ represents the grayscale loss of high-frequency fringe images, while $L_{phase}$ denotes the phase consistency loss of the one-period continuous phase.

The upcoming sections discuss the details of grayscale consistency loss, phase consistency loss, and the network architecture.

### 2.1. Grayscale Consistency Loss of High-Frequency Fringe Images

Inspired by photometric consistency in unsupervised deep learning for autonomous driving [28,29], grayscale consistency loss is used in predicting depth maps from fringe images of FPP. This loss measures the error between real high-frequency fringe images and synthesized ones. When the predicted depth map is correct, the synthesized fringe images will be very similar to the real ones.

The phase-shifting fringe images captured by the camera are:

(2) $$\left.I_{k}(i,j)=A(i,j)+B(i,j)cos[\Phi \left(i,j\right)+2\pi k/N\right]$$

where A(i,j) represents the background intensity, B(i,j) is the modulation, and Φ(i,j) denotes the absolute phase. The variable N indicates the phase-shifting steps; in this study, N=3 and k=0, 1, 2.

Background intensity A and modulation B are calculated as:

(3) $$A\left(i,j\right)=\frac{1}{3}\left[\;I_{0}\left(i,j\right)+I_{1}\left(i,j\right)+I_{2}\left(i,j\right)\right],$$

and

(4) $$B\left(i,j\right)=\frac{1}{3}\sqrt{{\left[2I_{0}\left(i,j\right)-I_{1}\left(i,j\right)-I_{2}\left(i,j\right)\right]}^{2}+3{\left[I_{1}\left(i,j\right)-I_{2}\left(i,j\right)\right]}^{2}}.$$

Depth map D, predicted by the network, is defined in the camera view. This depth map is converted into the point cloud defined in the camera’s 3D space, then transformed into the projector’s 3D space and projected on the projector plane. During this process, the projection flow is generated, which lies on the pixel grid of the camera. It associates the pixel grids of the camera with the corresponding floating point pixel coordinates in the projector pattern. The resolution of the projection flow is the same as that of depth map D, and it includes two channels along the row and col directions to locate a corresponding pixel coordinate in the projection pattern. Thus, for each point $p_{c}$ in the fringe images ($I_{0}$, $I_{1}$, and $I_{2}$), there is a corresponding point $p_{p}$ in the projector pattern. With this projection flow, the continuous phase map $\Phi_{cam}^{\prime }$ of the camera view is generated from the projector’s high-frequency continuous phase $\Phi_{proj}$. The size of $\Phi_{cam}^{\prime }$ is the same as that of $I_{0}$, $I_{1}$, and $I_{2}$; the phase value of the point $p_{c}$ in $\Phi_{cam}^{\prime }$ is equal to the continuous phase value of $p_{p}$ in the projector pattern.

With $\Phi_{cam}^{\prime }$ and background light intensity A and modulation B, three fringe images $I_{k}^{\prime }\left(k=0,1,2\right)$ are synthesized as follows:

(5) $$\left.I_{k}^{\prime }(i,j)=A(i,j)+B(i,j)cos[\Phi_{cam}^{\prime }\left(i,j\right)+2\pi k/3\;\right].$$

The grayscale consistency loss is formulated as follows:

(6) $${L_{gray}}_{k}=\frac{1}{\left|V\right|}\;\sum_{p\in V}\left({\lambda_{1}\;\left\|\left.I_{k}\left(p\right)-I_{k}^{\prime }\left(p\right)\right\|\right.}_{1}+\lambda_{2}\frac{1-SSIM_{kk^{\prime }}(p)}{2}\right),$$

and

(7) $$L_{gray}=\frac{1}{3}\sum_{k=0}^{2}{L_{gray}}_{k}.$$

where V represents the set of valid points with modulation greater than a threshold. These excluded invalid points are usually located in the background, shadow, and low-reflectivity areas. The number of points in V is denoted by |V|, where ${\left\|I_{k}\left(p\right)-I_{k}^{\prime }\left(p\right)\right\|}_{1}$ directly measures the differences between these two images. The item of $SSIM_{kk^{\prime }}$ is the structural similarity between real fringe images $I_{k}$ and synthesized ones $I_{k}^{\prime }$, which is formulated as follows [30]:

(8) $$SSIM(I_{k},I_{k}^{\prime })=\frac{\left(2\mu_{I_{k}}\mu_{I_{k}^{\prime }}+C_{1}\right)\left(2\sigma_{I_{k}I_{k}^{\prime }}+C_{2}\right)}{\left(\mu_{I_{k}}^{2}+\mu_{I_{k}^{\prime }}^{2}+C_{1}\right)\left(\sigma_{I_{k}}^{2}+\sigma_{I_{k}^{\prime }}^{2}+C_{2}\right)},$$

where $I_{k}$ and $I_{k}^{\prime }$ represent the two images; $\mu_{I_{k}}$ and $\mu_{I_{k}^{\prime }}$ are the mean values of $I_{k}$ and $I_{k}^{\prime }$; $\sigma_{I_{k}}$ and $\sigma_{I_{k}^{\prime }}$ are the standard deviations of $I_{k}$ and $I_{k}^{\prime }$; $\sigma_{I_{k}I_{k}^{\prime }}$ is the covariance of $I_{k}$ and $I_{k}^{\prime }$; and $c_{1}$ and $c_{2}$ are constants used for stability in computation. Here, $c_{1}$ is set to 0.49 and $c_{2}$ is set to 4.41, according to [30]. The fringe image exhibits periodic structures. The second term on the right side of Equation (6) could help the network learn features of this periodic structure. We follow the works in [28,31,32,33] and set $\lambda_{1}$ to 0.15 and $\lambda_{2}$ to 0.85.

**Figure 2:**
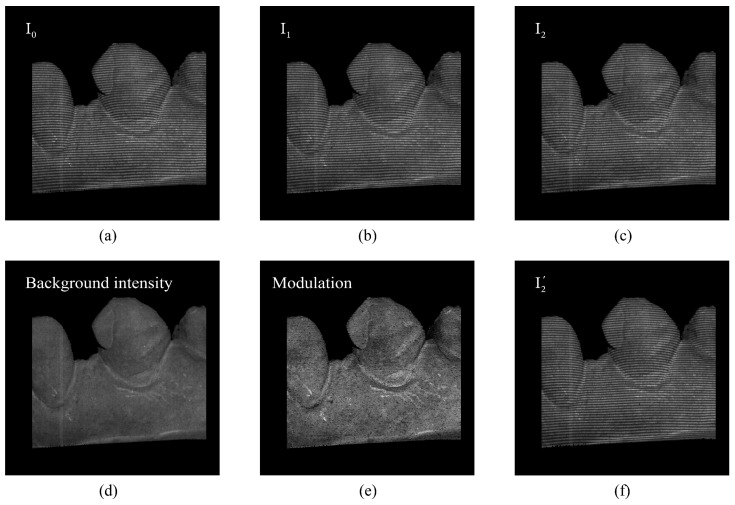
Comparison of the real fringe image and the synthesized one. (**a**–**c**) are the three-step phase-shifting fringe images. (**d**,**e**) show the background intensity A and the modulation B. (**f**) is the synthesized fringe image corresponding to the fringe image in (**c**). The SSIM and $L_{1}$ error between (**c**) and (**f**) are 96.22% and 2.272, respectively.

The fringe image in Figure 2f is synthesized according to Equation (5), where the phase map $\Phi_{cam}^{\prime }$ is generated using the depth map with a four-frequency temporal phase unwrapping (TPU) algorithm [28]. The SSIM between the two fringe images of Figure 2f and Figure 2c is 96.22%, and their $L_{1}$ error is 2.272. Therefore, the SSIM and $L_{1}$ error demonstrate the reliability of the proposed grayscale consistency. The difference between the real fringe image and the synthesized one can directly reflect the quality of the predicted depth map.

### 2.2. Phase Consistency Loss of One-Period Continuous Phase

**Figure 3:**
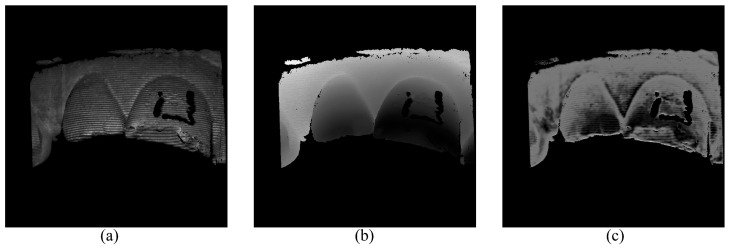
Wrong depth map predicted by the self-supervised network trained with only grayscale consistency loss of high-frequency fringe images. (**a**) One frame of the three-step phase-shifting fringe images. (**b**) The depth map with four-frequency TPU algorithm and triangulation. (**c**) The predicted depth map using the self-supervised network trained with only grayscale consistency loss of high-frequency fringe images.

In this study, we observe that relying solely on grayscale consistency loss of high-frequency fringe images does not produce effective training results [34]. For a point $p_{c}$ in camera view, its corresponding point $p_{p}$ should lie on the epipolar line in a projector pattern. The position and the phase value of point $p_{p}$ vary along the epipolar line with the change in the depth value of $p_{c}$. According to Equation (5), for two different points on the epipolar line, their phase values should be different, but the value of $I_{k}^{\prime }\left(k=0,1,2\right)$ at point $p_{c}$ may be the same for the two points because of the periodicity of the cosine function. Therefore, a point $p_{c}$ may correspond to different points on the epipolar line in projector patterns in grayscale consistency loss, meaning that the depth of point $p_{c}$ fails to converge to a unique value. As shown in Figure 3, the predicted depth map exhibits periodic fringe-like artifacts, which we attribute to depth ambiguity. We will elaborate on the comparison study of these losses in Section 3.4. Dual-frequency heterodyne fringe images are proposed to address this problem in the unsupervised depth estimation on simulated fringe images [27].

The one-period phase is used to eliminate the ambiguity to guide the network’s convergence. The phase of one-period phase maps is the absolute phase, which implicitly determines the 3D profile despite its poor accuracy. The wrapped phase of one-period fringe images is calculated as:

(9) $$\varphi_{1}=-\arctan\frac{\sum_{k=0}^{2}I_{k}\sin(2k\pi /3)}{\sum_{k=0}^{2}I_{k}\cos(2k\pi /3)}.$$

The wrapped phase $\varphi_{1}$ can be easily converted into an absolute phase $\Phi_{1}$ as:

(10) $$\Phi_{1}=\left\{\begin{array}{c} \varphi_{1},\;\varphi_{1}\geq 0\;\\ \varphi_{1}+2\pi ,\;\varphi_{1}<0.\; \end{array}\right.$$

The process discussed in Section 2.1 is utilized to synthesize a one-period continuous phase $\Phi_{1}^{\prime }$. The error between $\Phi_{1}$ and $\Phi_{1}^{\prime }$ is taken as the phase consistency supervisory signal. This loss item is as follows:

(11) $$L_{phase}=\gamma {\;L}_{abs}+\delta \;L_{gradient}.$$

where $L_{abs},$ presented in Equation (12), stands for the $L_{1}$ loss between the real one-period absolute phase $\Phi_{1}$ and the synthesized one-period absolute phase $\Phi_{1}^{\prime }$, while $L_{gradient},$ presented in Equation (13), denotes the $L_{1}$ loss between their gradients. We set γ=1 and δ=1 based on experiments.

(12) $$L_{abs}=\frac{1}{\left|V\right|}\sum_{p\in V}{\left\|\left.\Phi_{1}^{\prime }(p)-\Phi_{1}(p)\right\|\right.}_{1},$$

(13) $$L_{gradient}=\frac{1}{\left|V\right|}\sum_{p\in V}({\left\|\left.{\nabla \Phi }_{1_{\left(x\right)}}^{\prime }\left(p\right)-\nabla \Phi_{1_{\left(x\right)}}\left(p\right)\right\|\right.}_{1}+{\left\|\left.{\nabla \Phi }_{1_{\left(y\right)}}^{\prime }\left(p\right)-\nabla \Phi_{1_{\left(y\right)}}\left(p\right)\right\|\right.}_{1})\;.$$

Here, V represents the valid points as defined in Equation (6), while ∇ denotes the first derivative along spatial directions, and we calculate the gradients along both the *x* and *y* directions.

### 2.3. Network Architecture

**Figure 4:**
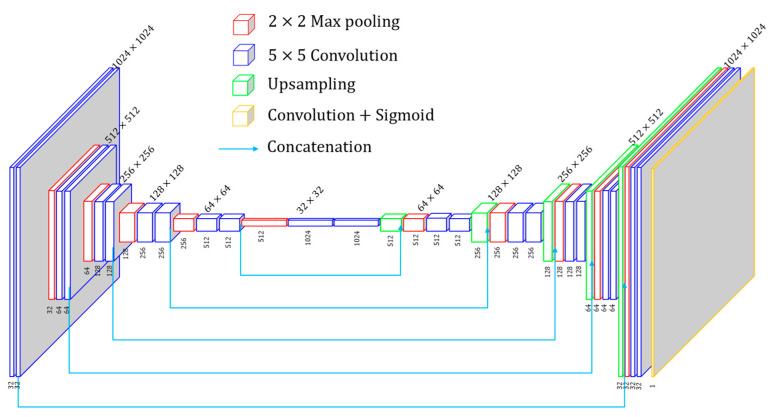
The proposed network architecture.

Previous fully supervised depth estimation for fringe projection profilometry [18] employed AEN, FCN, and UNet [35]. Results indicate that the UNet performs better. In simulated experiments, UNet also exhibits effective performance [23]. In our study, ERFNet [36], EESANet [37], and Unet are tried, and the results indicate that UNet performs the best. Therefore, UNet is chosen as the depth network. As shown in Figure 4, in our implementation, each encoder and decoder block adopt 5 × 5 kernels. The image size is reduced by half with every encoder block passed, while it is doubled with every decoder block passed. Finally, a 5 × 5 convolution layer is attached to the final layer of the last decoder block to transform the feature maps to the desired size of the depth map. According to comparative experiments, we find that limiting the depth range based on the camera’s workspace is more effective than the arbitrary depth range. The output of the UNet is passed through a sigmoid function so that the output values of the network lie within the interval (0,1). Three-step phase-shifting high-frequency fringe images serve as input for the depth network, and the output is a single-channel depth map with the exact resolution as the input. Next, the predicted depth values are applied to build the projection flow for the purpose of synthesizing the continuous phase and fringe images. Compared to a single fringe image input into the network, three frames of fringe images complement each other to provide more detailed information on the object’s surface.

## 3. Experiments and Results

Experiments were conducted on real FPP datasets to verify the effectiveness of the proposed method. These experiments included comparative experiments as well as ablation studies. In the comparative experiment, the proposed method was compared with the supervised method [18] and dual-frequency (DF-TPU) [9], with the depth of multi-frequency temporal phase unwrapping (MF-TPU) [8,38,39] as the ground truth. These comparisons were made under typical scenes, including smooth surfaces, abrupt shape change, image defocusing [40], low reflectivity, motion blur, and isolated objects.

### 3.1. Dataset

A handheld FPP system was used to collect data. The design working distance of this FPP system is 110 mm, the angle between the optical axis of the camera and the projector is 13 degrees, and the measurement volume is 12 mm×12 mm×10 mm. One CMOS camera of 1024 × 1024 pixels and a DLP projector of 684 × 608 pixels were used. During the process of handheld scanning, heavy noise caused by motion blur, projection defocusing, and imaging defocusing was inevitably introduced into most samples of the training dataset. Heavy noise poses a challenge in terms of depth estimation.

Four-frequency (period-number of 1, 4, 16, and 64) three-step phase-shifting fringe patterns were projected to reconstruct the ground-truth depth maps. Only a one-period phase map and three frames of 64-period fringe images were used to train the network, and three frames of 64-period fringe images were used to test the performance of the network.

**Figure 5:**
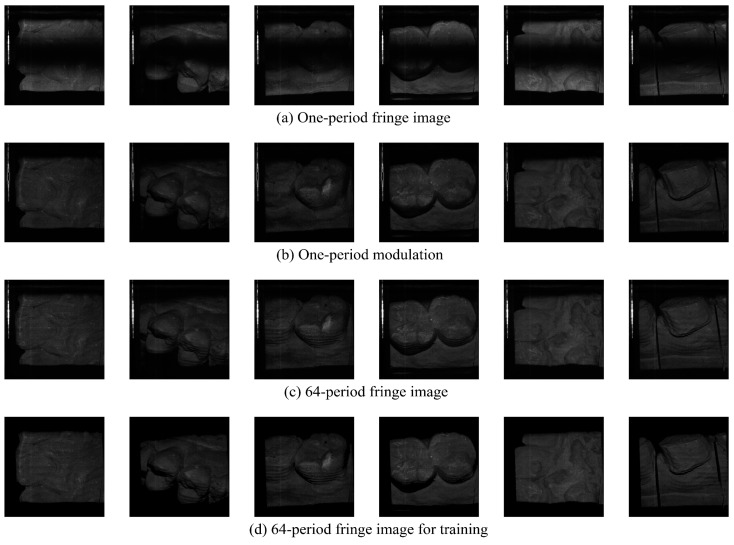
Examples of the dataset. The input image size of the neural network is 1024 × 1024 pixels. (**a**) shows one-period fringe images. (**b**) illustrates one-period modulation maps. (**c**) shows original 64-period fringe images, and (**d**) displays 64-period fringe images for training after preprocessing.

The training dataset contained 1480 groups of fringe images from seven dental models, the validation dataset contained 284 groups of fringe images from a single dental model, and the test dataset contained 506 groups of fringe images from two dental models. The data collection was accomplished within 10 min. Figure 5 demonstrates some examples of the collected data.

Figure 5 shows some typical examples of the collected dataset. Please note that non-ideal data account for more than 50% of the collected data. Non-ideal data include motion blur, low surface reflectivity, image defocusing, fringe discontinuity, and overexposure. These complex factors lower the quality of fringe images and pose a challenge to the training of the depth prediction network and the robustness of deep-learning-based depth prediction networks.

Please note that the camera of the handheld FPP system was custom-designed instead of a commercial off-the-shelf product. Fringe images were converted from the RAW data of the CMOS sensor. Except for a fixed gain parameter and automatic black level during this conversion, no other image signal processing tasks were performed, such as exposure correction, denoising, sharpening, or gamma correction. Therefore, the intensity value of the fringe images from our FPP system was relatively low.

During data preprocessing, the invalid points and background points were removed according to the modulation threshold. The modulation threshold was set to 14 for one-period fringe images. Next, morphology operations (erosion followed by dilation) were carried out to eliminate noise points at the edges of objects. At last, areas with less than one percent of the total number of pixels were removed.

### 3.2. Network Implementation

The network and the weakly-supervised framework were implemented using PyTorch. The training and inference were performed on an NVIDIA Titan RTX. An ADAM optimizer with a momentum of 0.9 and a weight decay of ${1\times 10}^{-4}$ was adopted. During training, the batch size was 2 and the initial learning rate was ${5\times 10}^{-5}$. The network was trained using 100 epochs for 30 h. The dimensions of the input images and the output depth map were 1024 × 1024 pixels.

The network of the comparative supervised method [18] was implemented by us. The hyperparameters and the training epochs were also set according to [18].

### 3.3. Comparison Results

During evaluation, we measured the frames per second (FPS) of the supervised network and ours on the same training device. The elapsed time per frame started from data being uploaded to the GPU and ended with the download of predicted data to the CPU. Finally, we calculated the mean elapsed time to derive the FPS. The FPS of the supervised network was 15.69, whereas the FPS of the proposed network was 4.92. Additionally, the parameter size of the supervised model was 147.98 MB, while that of our model was 399.80 MB.

**Figure 6:**
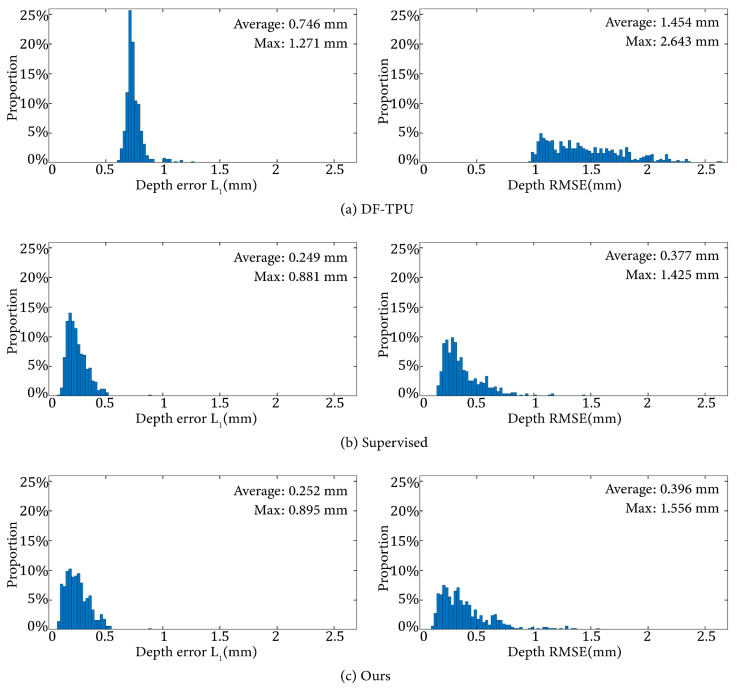
Distributions of the depth error $L_{1}$ and RMSE from the results of the three methods.

The $L_{1}$ norm and RMSE of the depth error were used to evaluate the quantitative performance of various methods. Table 1 lists the average evaluation metrics of these methods on the 506 samples of the test dataset. Figure 6 shows the distribution of $L_{1}$ and RMSE of the depth error corresponding to Table 1.

It can be observed that DF-TPU produced a larger mean $L_{1}$ and RMSE and a wider distribution of RMSE than the supervised method and ours. Our method and the supervised method showed similar performances in terms of the mean and distribution of $L_{1}$ and RMSE.

**Figure 7:**
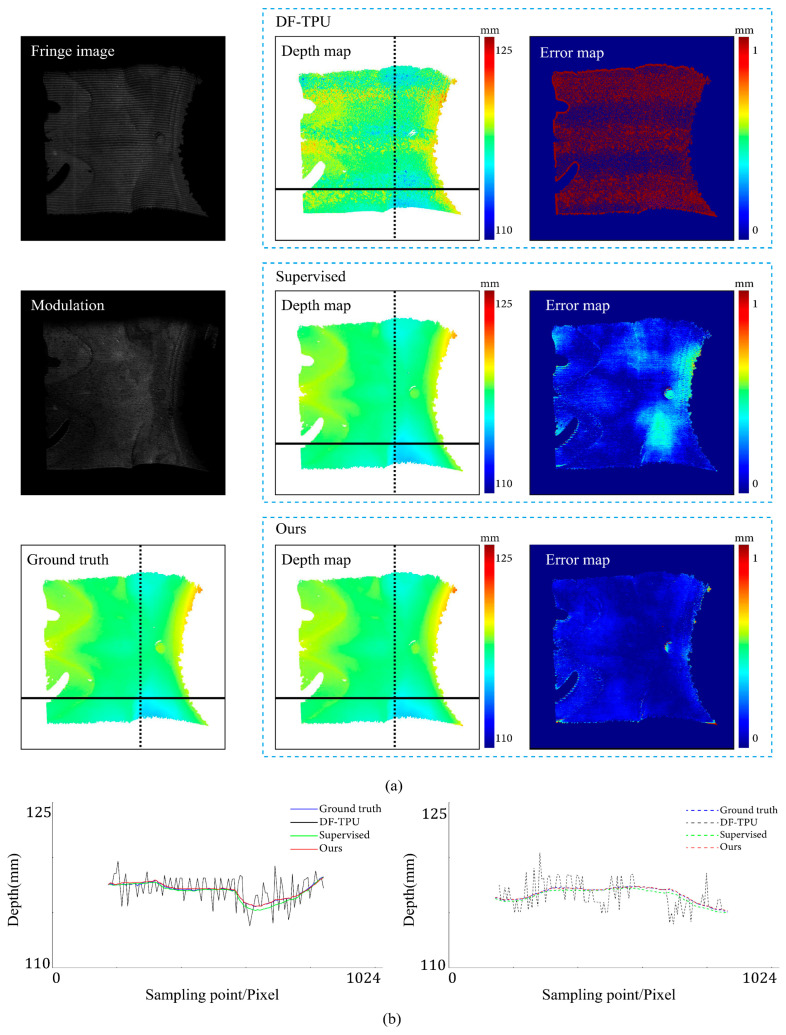
Comparison results of the smooth surface object. (**a**) shows the predicted depth map and the error map. (**b**) depicts the depth values distribution of two random horizontal and vertical pixel coordinate lines in the four depth maps of (**a**).

**Figure 8:**
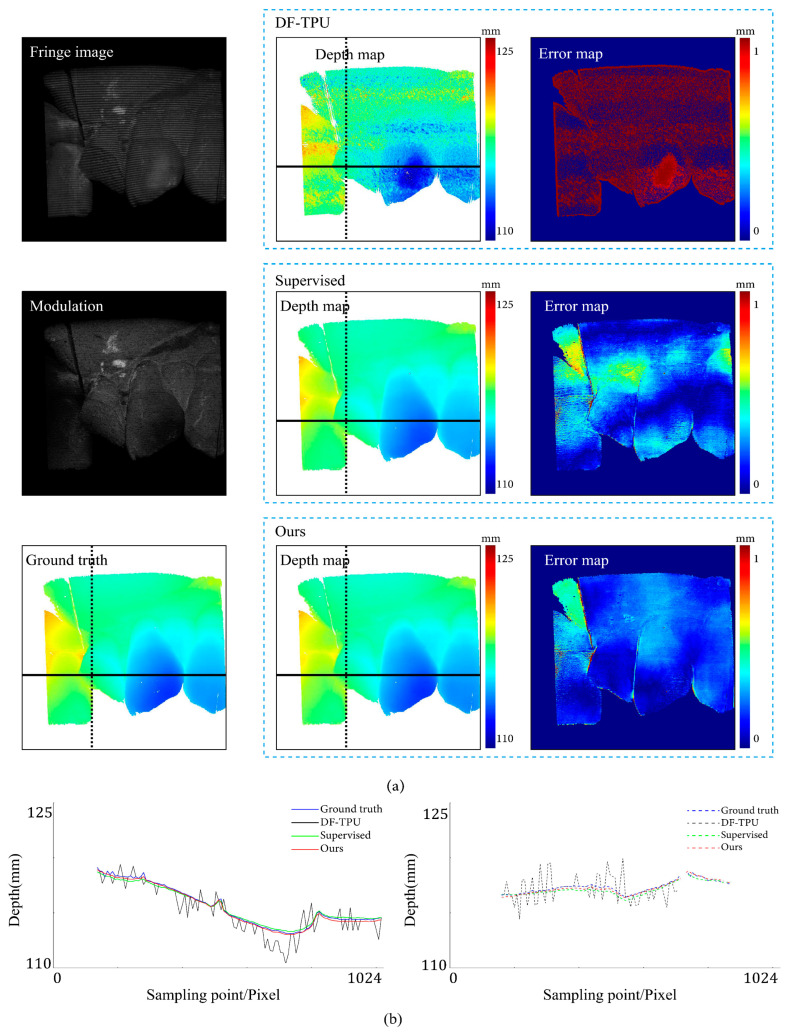
Comparison results of an object with abrupt shape change. (**a**) shows the predicted depth map and the error map. (**b**) depicts the depth values distribution of two random horizontal and vertical pixel coordinate lines in the four depth maps of (**a**).

**Figure 9:**
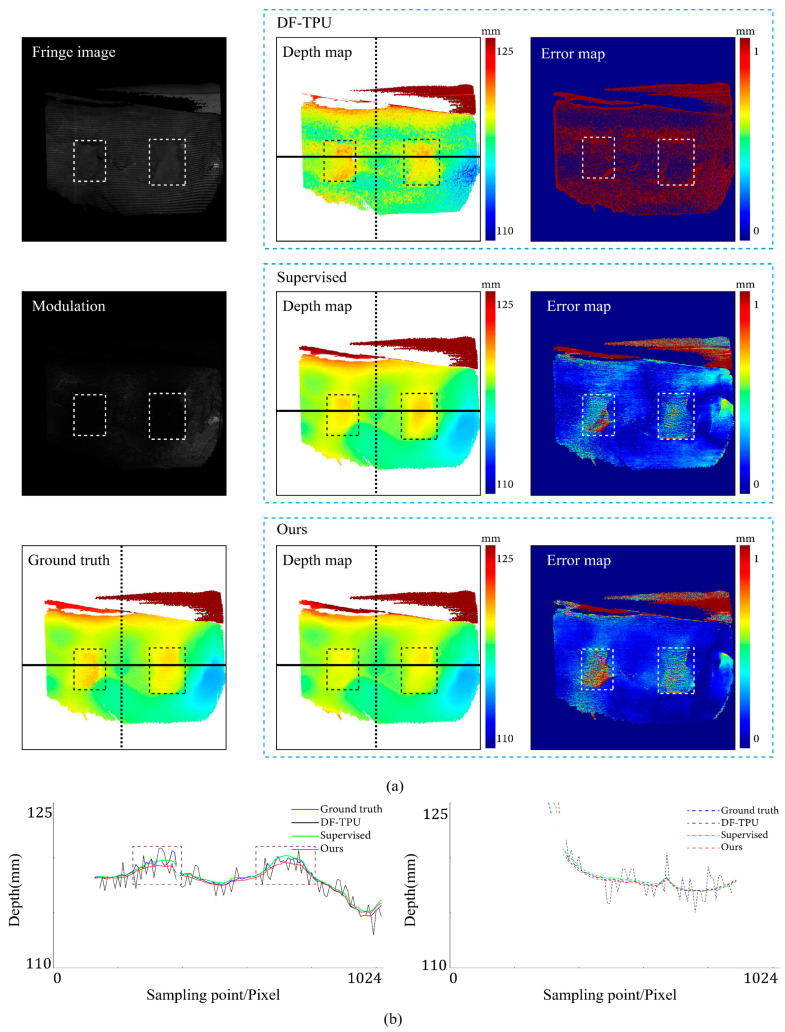
Comparison results for the scene of image-defocusing. The dotted boxes highlight the defocusing areas, and their colors are selected to improve visualization. (**a**) shows the predicted depth map and the error map. (**b**) depicts the depth values distribution of two random horizontal and vertical pixel coordinate lines in the four depth maps of (**a**).

**Figure 10:**
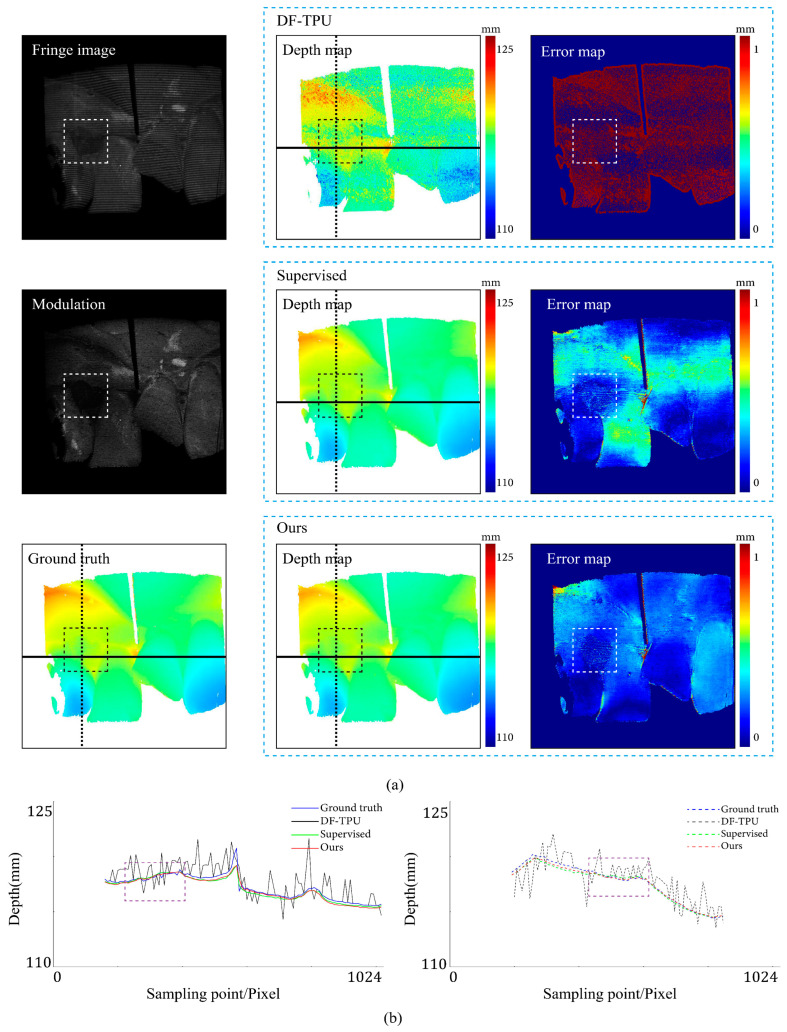
Comparison results for the scene of low surface reflectivity. The dotted boxes highlight the areas with low reflectivity, and their colors are selected to improve visualization. (**a**) shows the predicted depth map and the error map. (**b**) depicts the depth values distribution of two random horizontal and vertical pixel coordinate lines in the four depth maps of (**a**).

**Figure 11:**
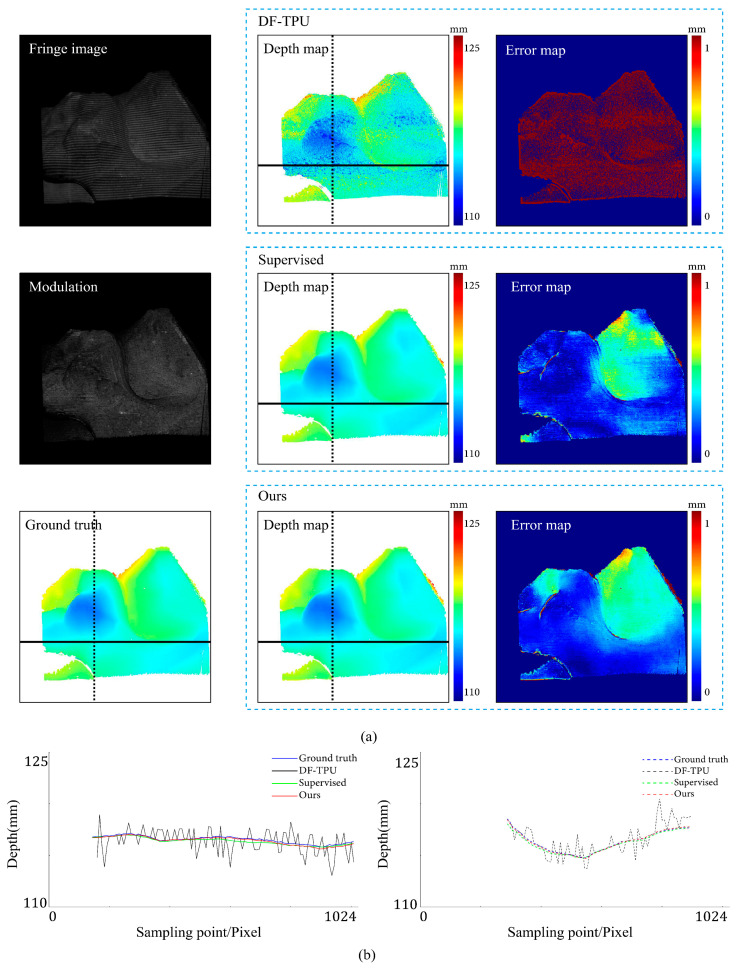
Comparison results for the scene of motion blur. (**a**) shows the predicted depth map and the error map. (**b**) depicts the depth values distribution of two random horizontal and vertical pixel coordinate lines in the four depth maps of (**a**).

**Figure 12:**
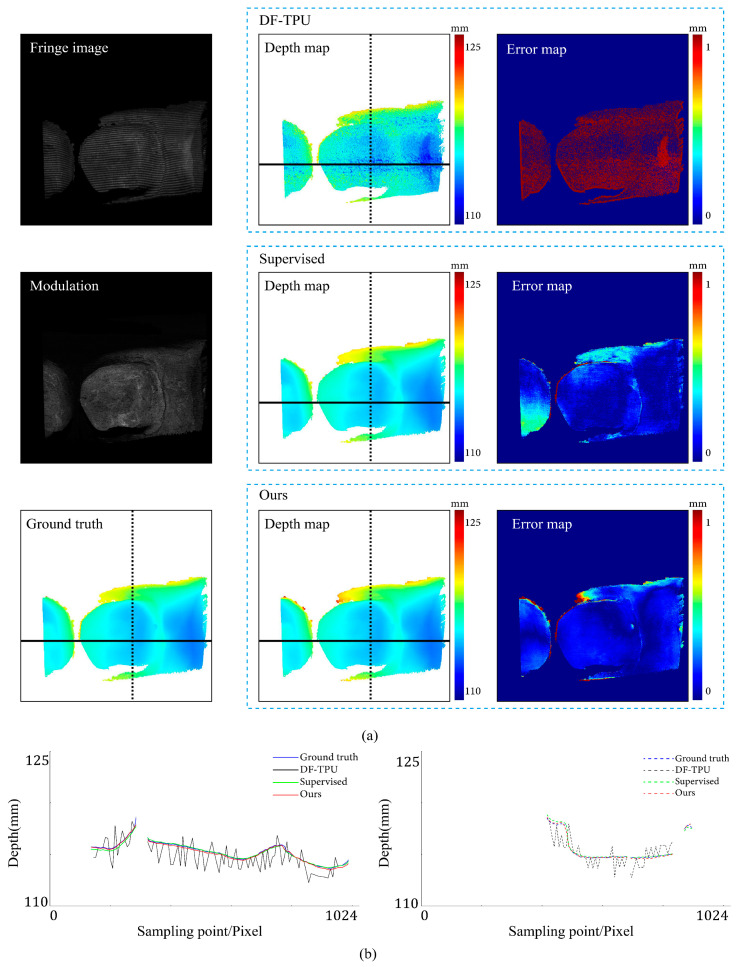
Comparison results for the scene of isolated objects. (**a**) shows the predicted depth map and the error map. (**b**) depicts the depth values distribution of two random horizontal and vertical pixel coordinate lines in the four depth maps of (**a**).

Figure 7, Figure 8, Figure 9, Figure 10, Figure 11 and Figure 12 illustrate the predicted results of the six representative scenes in the test dataset. In each figure, group (a) shows the predicted depth map and the error map, and group (b) depicts the similarity between the ground truth and the predicted depth map. This similarity is evaluated by comparing the depth values distribution of two random horizontal and vertical pixel coordinate lines. As shown in Figure 7, Figure 8, Figure 9, Figure 10, Figure 11 and Figure 12, the solid and dotted lines represent the horizontal and vertical indicator lines, respectively. All the ground-truth depth maps were produced using the hierarchical MF-TPU algorithm and triangulation.

Figure 7 shows the results of the three methods in the scene of a smooth surface. Compared to the supervised method, our method generated a more uniform distribution of depth value errors in the error map. The lines of depth value in Figure 7b also verify this observation. Compared to the other two lines, the red line produced with our methods is more consistent with the ground truth line.

Figure 8 shows the results of the three methods in a scene with abrupt shape change. Our method and the supervised method had large errors in the local area near the shape edge. Compared to the supervised method, our method generated a relatively more uniform distribution of depth value errors in the error map. In Figure 8b, the depth value line of our method is very close to that of the supervised method.

Figure 9 shows the results of the three methods in a scene with image defocusing. The rectangular boxes indicate the defocusing areas. Please note that the ground-truth depth values of defocusing areas showed significant fluctuations. These fluctuations mean that the ground truth had errors. Our method and the supervised method predicted depth values with errors in defocusing areas. Our method generated a slightly worse distribution of depth value errors than the supervised method. As can be seen in the left part of Figure 9b, in defocusing areas, the red line (results of our method) deviates from the ground truth more significantly than the green line (results of the supervised method).

Figure 10 shows the results of the three methods in a scene with low reflectivity. Compared to the supervised method, our method generated a more uniform distribution of depth value errors in the error map. The rectangular box indicates the area with low reflectivity. Our method and the supervised method predicted the depth values of small errors in this area. In the left part of Figure 10b, within the rectangular box representing the low reflectivity area, both the red line (results of our method) and the green line (results of the supervised method) show slight deviations from the ground truth. The ground truth depth values in this area exhibited minor fluctuations attributed to poor fringe quality in low-reflectivity areas. In the right part of Figure 10b, within the rectangular box, the red line closely aligns with the ground truth, displaying closer proximity to the ground truth than the green line (results of the supervised method).

Figure 11 shows the results of the three methods in a scene with motion blur. From the modulation map, it can be observed that there are evident zig-zag artifacts, indicating the presence of motion blur in the scene. Compared to the supervised method, our method exhibited more minor depth value errors in the internal edge area of the object. In addition, our method did not exhibit significant depth value errors throughout the entire image. In Figure 11b, the depth line of our method closely matches the ground truth, performing better than the supervised method.

Figure 12 shows the results of the three methods in a scene with isolated objects. From the image, it is evident that the objects were separated. Based on the error map, our method exhibited a more uniform distribution of depth value errors than the supervised method. In the left part of Figure 12b, for the object on the left side, the red line (results of our method) closely aligns with the ground truth, while the green line significantly deviates from the ground truth; for the object on the right side, our method is close to the ground truth, but the supervised method is more accurate. In the right part of Figure 12b, both our method and the supervised method are very close to the ground truth, and at the upper edge of the object, our method performed better.

In summary, DF-TPU produced erroneous periodic structures in the depth maps, as shown in the error maps of Figure 7, Figure 8, Figure 9, Figure 10, Figure 11 and Figure 12. The dramatic ups and downs of the depth curves also demonstrate this. Among the six representative scenes, DF-TPU generated depth maps with drastic changes in depth values. The errors in these depth maps are too large to be used for 3D reconstruction. Our method performed equally or better than the supervised method in the representative scenes, except for image defocusing. Due to the presence of many defocused areas in the training, validation, and test datasets, our method is slightly worse than the supervised method in the average quantitative indicator of Table 1.

### 3.4. Ablation Study of Proposed Phase Consistency Loss

An ablation study on the same dataset was conducted to verify the effectiveness of the proposed phase consistency loss. We trained the network with only grayscale consistency loss, only phase consistency loss, and a combination of these two losses. The results demonstrate the contribution of the proposed items to the overall performance of the network. The specific items included (#1) only grayscale consistency loss; (#2) only phase consistency loss; and (#3) a combination of grayscale consistency loss and phase consistency loss.

The three items were utilized on the same training dataset and examined on the same test dataset. We trained the three networks for 100 epochs with the same super parameters, where the batch size was set to 2 and the starting learning rate was set to ${5\times 10}^{-5}$. The evaluation metrics are recorded in Table 2. The ablation experiment verified the necessity of the proposed phase consistency loss and the effectiveness of a combination of the two losses.

**Figure 13:**
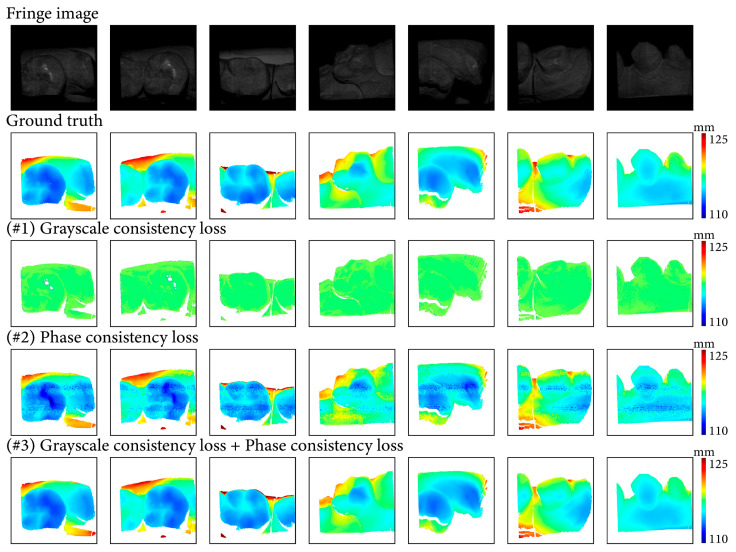
Results of the ablation experiment on proposed items.

Figure 13 illustrates the depth maps with the three items. In these scenes, the depth maps of #1 deviate from ground truth, and the indicators in Table 2 also support it. In Table 2, the $L_{1}$ error and RMSE illustrate that network trained with only grayscale consistency loss could not output a correct depth map. The depth maps of #2 provide absolute depth scale information despite a large number of depth errors, and the indicators of depth errors in Table 2 demonstrate the effectiveness of phase consistency loss. At the end, the depth maps of #3 are the results of the network trained with a combination of the proposed two losses, which is very close to the ground truth. In Table 2, the $L_{1}$ error and RMSE of #3 are further reduced compared to #2.

### 3.5. Ablation Study of The Loss Function

The effectiveness of each loss item of the proposed loss function was verified by an ablation experiment on the same training dataset. Seven combinations of different loss items were tested in this ablation experiment. The seven combinations included (#1) only $L_{abs}$ as the loss function, (#2) only $L_{gradient}$ as the loss function, (#3) only $L_{gray}$ as the loss function, (#4) $L_{abs}+L_{gradient}$ as the loss function, (#5) $L_{abs}$ + $L_{gray}$ as the loss function, (#6) $L_{gradient}$ + $L_{gray}$ as the loss function, and (#7) $L_{phase}$ + $L_{gray}$ as the loss function. The #7 combination was the loss function of the proposed weakly-supervised depth estimation network.

The seven networks corresponding to the seven loss functions were trained on the same training dataset with the same super parameters, including a batch size of 2, a starting learning rate of ${5\times 10}^{-5}$ s, and 100 training epochs. Table 3 illustrates the evaluation metrics of the seven networks on the same test dataset. This ablation experiment verified the effectiveness of the loss function of our method.

**Figure 14:**
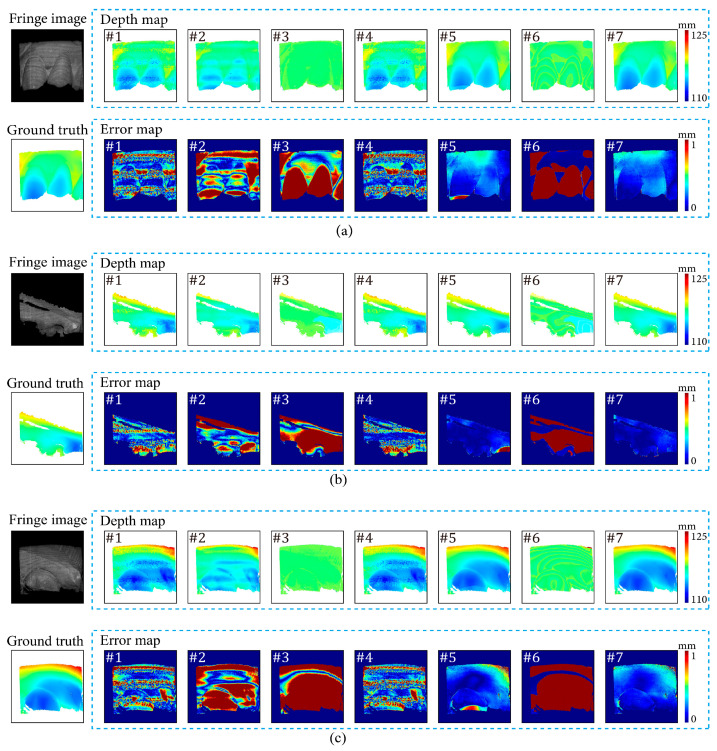
Results of the ablation experiment. The contrast of fringe images is enhanced for better visualization. (**a**) shows the results of seven networks in the scene with abrupt depth changes and low reflectivity, (**b**) shows the results of seven networks in the scene containing smooth surfaces, and (**c**) shows the results of seven networks in the scene containing smooth surfaces and abrupt depth changes.

Figure 14 illustrates the depth maps and error maps with the seven implementations. In all the scenes, depth maps of #1 to #4 as well as #6 exhibit significant deviations from the ground truth. Among the remaining two implementations of #5 and #7, #5 exhibits noticeable prediction errors in some local regions, while no fringe-like structures are present within these regions. The proposed method (#7) attains the highest performance.

### 3.6. 3D Reconstruction

**Figure 15:**
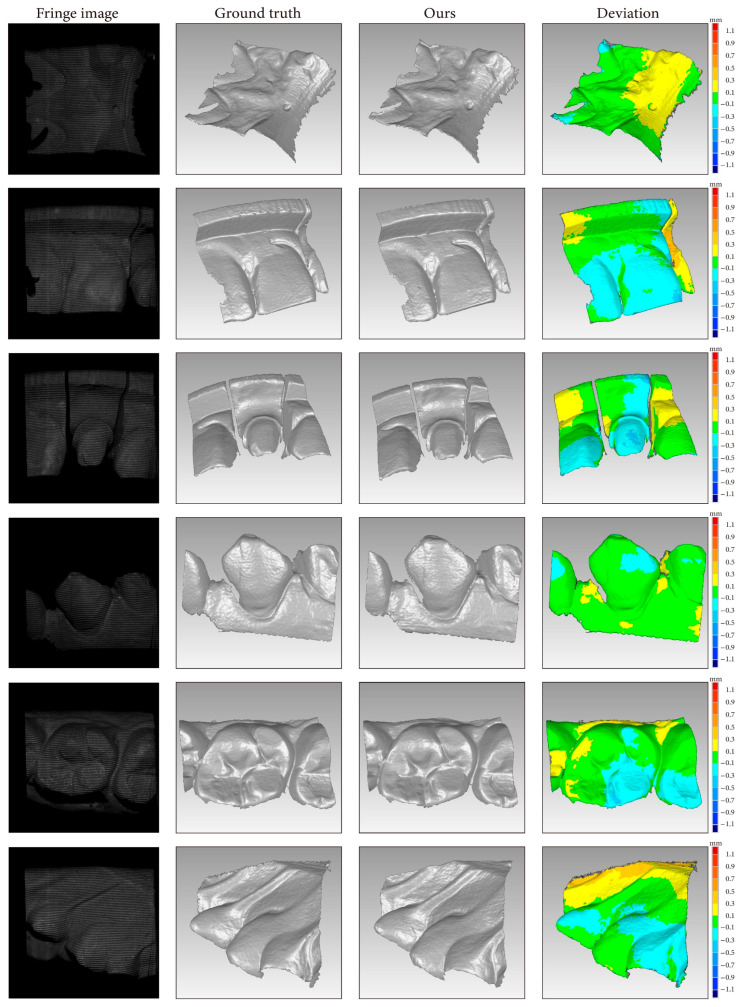
Point clouds reconstructed with the proposed method. The depth interval for the FPP system was 110–125 mm.

Point clouds reconstructed from the depth maps produced by the weakly supervised method are shown in Figure 15. The deviation map after point cloud alignment shows that the point cloud reconstructed by our method had local errors. The depth RMSE of the six unseen scenes was 0.12 mm. The depth interval of the FPP system was 110–125 mm. The average depth RMSE was approximately 0.096–0.109% of the depth interval.

### 3.7. Comparison on 16-Period Fringe Images

We experimented to compare the performance of the supervised network and ours on 16-period fringe images. The depth obtained from MF-TPU of three-frequency (period-numbers of 1, 4, and 16) three-step phase-shifting fringe images was used as the ground truth. The split of training, validation, and test datasets was the same as that in Section 3.1, and the hyperparameters for training the supervised network and ours were identical to those in Section 3.2. The evaluation metrics are listed in Table 4.

**Figure 16:**
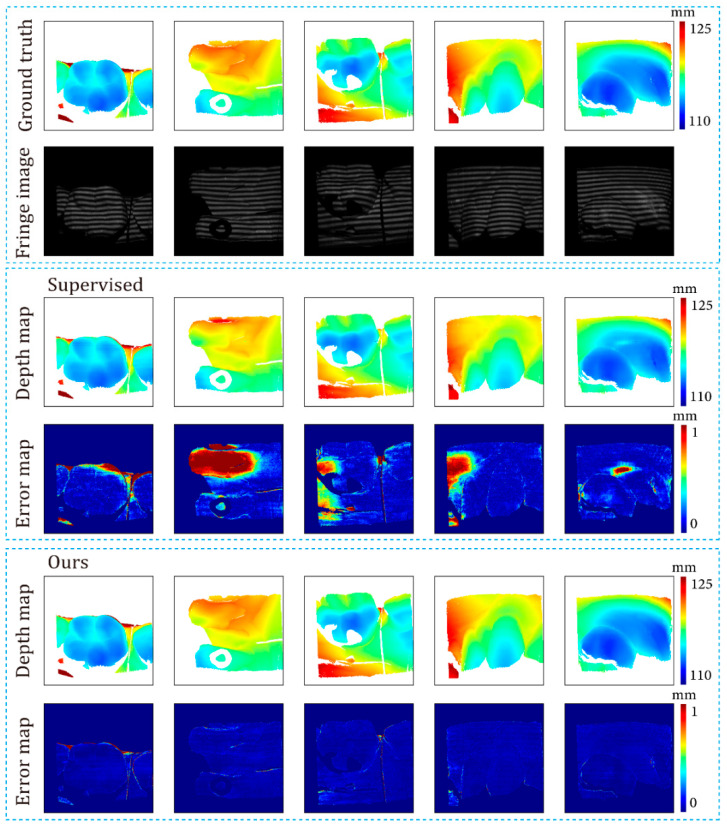
Results of the supervised method and proposed method on 16-period fringe images.

Table 4 and Figure 16 demonstrate that our method outperformed the supervised method on 16-period fringe images.

### 3.8. Comparison on Datasets of Various Noise Levels

We simulated a noise-free dataset, and various levels of noise were introduced to this noise-free dataset. The supervised network and ours were trained and tested on these datasets.

With the depth of MF-TPU, the relative pose between the camera and projector, and the continuous phase $\Phi_{proj}$ of 64-period patterns on the projector plane, one continuous phase map $\Phi_{cam}^{\prime }$ of 64-period in the camera view was generated. By setting the background intensity A to a constant value of 120 and the modulation B to a constant value of 100, the continuous phase $\Phi_{cam}^{\prime }$ was modulated into three frames of 64-period fringe images according to Equation (5). Three frames of one-period fringe images were synthesized using the same approaches. These two-frequency (period-numbers of 1 and 64) three-step phase-shifting fringes were noise-free. Then, Gaussian white noise with signal-to-noise ratios (SNRs) of 20, 25, 30, and 35 was added to the noise-free fringe images separately. Finally, we obtained multiple datasets of fringe images with various levels of noise.

The split of the training, validation, and test datasets was the same as that in Section 3.1, and the hyperparameters for training the supervised network and ours were identical to those in Section 3.2.

**Figure 17:**
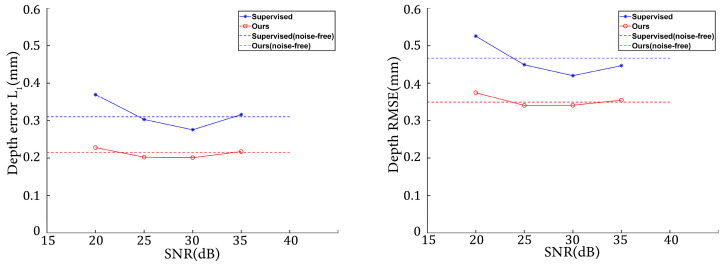
Evaluation metrics on datasets with different levels of noise.

**Figure 18:**
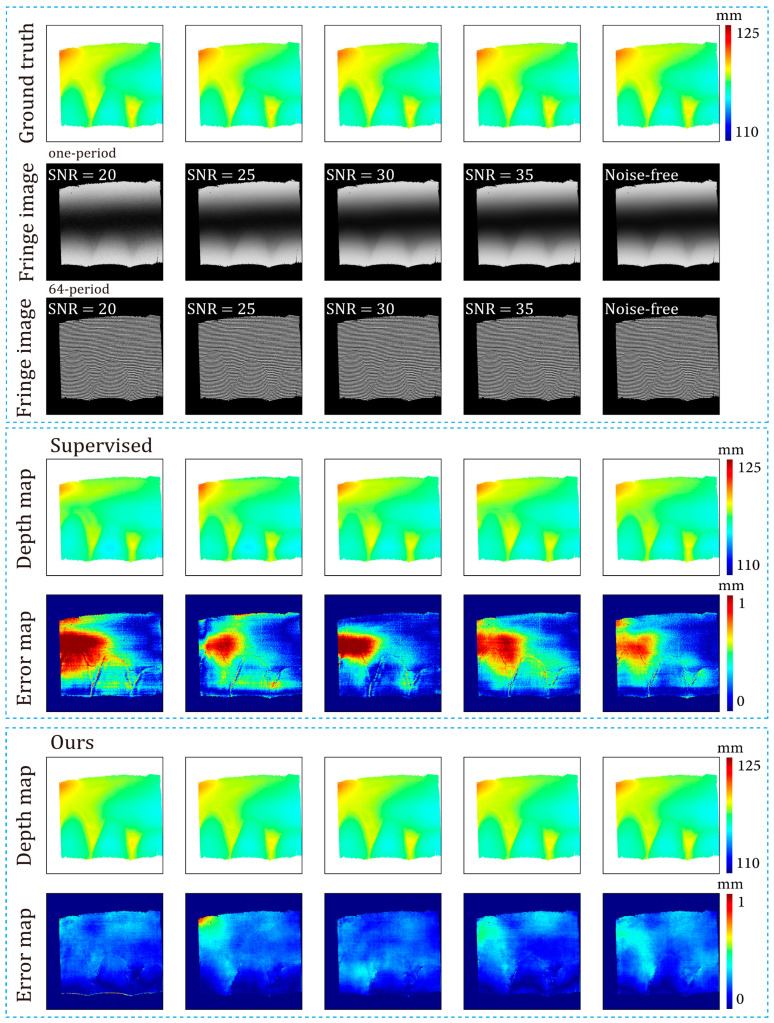
Predicted depth maps of the both supervised method and the proposed method and datasets with different levels of noise.

Figure 17 illustrates the variations in depth $L_{1}$ error and depth RMSE as the noise level changed. Combining Table 1 and Figure 17, it can be observed that our method showed better robustness to various levels of noise than the supervised method. Note that both the supervised method and ours demonstrated better performance on simulated datasets with SNRs of 25 and 30. This may be because adding noise amounts to an operation of dataset augmentation. In deep learning, proper data augmentation can improve the generalization of the model. Figure 18 presents the fringe images at various noise levels, along with the corresponding depth maps predicted by the networks. Note that the noise of the dataset in Section 3.3 was heavier than that of the simulated datasets in this Section. Figure 17 and Figure 18 show that our method overperformed the supervised method on the less noisy simulated datasets.

## 4. Discussion

### 4.1. Efficiency

Measurement speed is one of the core goals of various FPP approaches, assuming that *N*-step (N ≥3) phase-shifting fringe patterns are employed. According to Equations (2), (9), and (10), when the period-number is set to one, the absolute phase can be directly obtained from N frames of fringe images, but its accuracy is relatively low. To improve phase accuracy, we need to increase the period-numbers of fringe images. However, when the period-number is greater than one, due to the periodic nature of the cosine function in Equation (2), the absolute phase cannot be directly obtained from the arctangent function in Equation (9). Instead, only a wrapped phase can be obtained. Therefore, in traditional methods, we typically require 2×N (N≥3) frames of fringe images for 3D reconstruction. Traditional dual-frequency TPU methods generally need 2×N frames of fringe images for one 3D reconstruction. This number is increased to 3×N or 4×N when high accuracy is required. For example, when 64-period fringe patterns are employed, traditional FPP approaches usually capture 4×N frames of fringe images for one 3D reconstruction. For the proposed method, the required number of fringe images during the training stage is 2×N when the 64-period fringe patterns are employed. During the inference stage, the required number of fringe images for one 3D reconstruction is N. Compared with traditional multi-frequency FPP approaches, the efficiency improvement rate of the proposed methods is:

(14) $$\eta =\frac{n\times N-N}{n\times N}(n=2,\;3,\;4,\ldots ).$$

When N = 3 and n = 2, η=50%; N = 3 and n = 4, η=75%. The proposed method is at least 50% more efficient than conventional non-DL-based multi-frequency FPP methods.

The efficiency improvement rate of the fully supervised method is at least (2×3−1)/(2×3)≈ 83.33%. However, the difficulty of obtaining labeled depth data has hindered the application of this approach. The efficiency improvement rate of the unsupervised approach [27] is at least (2×3−2)/(2×3)≈66.67%, but its feasibility has only been verified with simulation data on a simplified FPP model; real FPP systems and application scenarios are more complex.

### 4.2. Accuracy

On the real dataset of 64-period fringe images, the proposed method showed competitive depth accuracy (depth error $L_{1}$ increased by 1.2% and depth RMSE increased by 5.0%) to the fully supervised method, and significantly higher accuracy (depth error $L_{1}$ decreased by 66.2% and depth RMSE decreased by 72.8%) than conventional DF-TPU. On unseen test objects, the average depth deviation was 0.12 mm.

This study used a handheld FPP system to collect data for training and testing. There was inevitable motion blur, projection defocus, and imaging defocus in most of the data. These factors brought significant noise to the 3D reconstruction. On the test dataset including 506 samples, the mean depth RMSE of the proposed method was 0.32–0.36% of the depth interval and 2.64% of the system depth range. For unseen scenes without imaging defocus, the mean depth RMSE of the proposed method was 0.096–0.109% of the depth interval of 0.80% of the depth range. If high-quality fringe images were captured, the deviation between the results of the proposed method and those of the four-frequency TPU could be reduced further.

### 4.3. Future Work

It was observed from our experiments that image defocusing impairs the depth estimation; excluding these defocusing areas from the fringe images may help to better the results. We will address this topic in our future work.

Additionally, for the handheld FPP system we used to collect the data, the measurement volume was 12 mm × 12 mm × 10 mm, the working distance was 110 mm, and the angle between the optical axis of the camera and the projector was 13 degrees. These specifications theoretically limited the depth accuracy of this FPP system. Future work will be conducted to verify the performance of the proposed method with data from different FPP systems.

## 5. Conclusions

A weakly supervised depth estimation technique for 3D reconstruction using high-frequency fringe images is presented in this study. The suggested methodology differs from the fully supervised deep learning method in that it does not need a depth map as a label. The potential application situations for deep-learning-based FPP depth estimation algorithms have been greatly expanded by the proposed method. Efficiency in terms of the number of fringe patterns was increased by 50% compared to conventional dual-frequency FPP approaches. The experimental results verify that the suggested method achieves competitive accuracy to fully supervised methods and doubles the maximum period-number of the conventional dual-frequency PSP, in addition to significantly improving the accuracy.

## Figures and Tables

**Table 1:** Average evaluation metrics of the three methods on the test dataset.

Method	$Depth\;Error\;L_{1}\;(mm)↓$	Depth RMSE (mm)↓
DF-TPU	0.746	1.454
Supervised	0.249	0.377
Ours	0.252	0.396

Note: The measurement depth interval of the FPP system was 110–125 mm.

**Table 2:** Comparison results of the proposed three items on the test dataset.

ID	Loss Function	$Depth\;Error\;L_{1}\;(mm)↓$	Depth RMSE (mm)↓
#1	$L_{gray}$	1.908	2.288
#2	$L_{phase}$	0.467	0.645
#3	$L_{phase}+L_{gray}$	0.252	0.396

**Table 3:** Comparison results of the seven loss combinations on the test dataset.

ID	Loss Function	$Depth\;Error\;L_{1}\;(mm)↓$	Depth RMSE (mm)↓
#1	$L_{abs}$	0.463	0.634
#2	$L_{gradient}$	0.850	1.056
#3	$L_{gray}$	1.908	2.288
#4	$L_{abs}+L_{gradient}$	0.467	0.645
#5	$L_{abs}+L_{gray}$	0.253	0.407
#6	$L_{gradient}+L_{gray}$	1.754	2.077
#7	$L_{abs}+L_{gradient}+L_{gray}$	0.252	0.396

**Table 4:** Average evaluation metrics of models trained and tested on 16-period fringe images.

Method	$Depth\;Error\;L_{1}\;(mm)↓$	Depth RMSE (mm)↓
Supervised	0.148	0.298
Ours	0.073	0.277

## Data Availability

Dataset available on request from the authors.
